# ^18^F-VC701-PET and MRI in the in vivo neuroinflammation assessment of a mouse model of multiple sclerosis

**DOI:** 10.1186/s12974-017-1044-x

**Published:** 2018-02-05

**Authors:** Sara Belloli, Lucia Zanotti, Valentina Murtaj, Cristina Mazzon, Giuseppe Di Grigoli, Cristina Monterisi, Valeria Masiello, Leonardo Iaccarino, Andrea Cappelli, Pietro Luigi Poliani, Letterio Salvatore Politi, Rosa Maria Moresco

**Affiliations:** 1 IBFM-CNR, Segrate, Italy; 2 Experimental Imaging Center, IRCCS San Raffaele Scientific Institute, Milan, Italy; 3 Milan Center for Neuroscience (NeuroMI) University of Milano-Bicocca, Milan, Italy; 4 Unit of Immunogenetics, Leukemia Genomics and Immunobiology, IRCCS San Raffaele Scientific Institute, Milan, Italy; 5 PhD Program in Neuroscience, University of Milan-Bicocca, Monza, Italy; 6 Department of Medicine and Surgery, University of Milano-Bicocca, Via Cadore 48, Monza, 20900 Italy; 7 Humanitas Clinical and Research Centre, Rozzano, Italy; 8 Biomedical Sciences Department, University of Padua, Padua, Italy; 9 Vita-Salute San Raffaele University and In Vivo Human Molecular and Structural Neuroimaging Unit, Division of Neuroscience, IRCCS San Raffaele Scientific Institute, Milan, Italy; 10 Department of Biotechnology, Chemistry and Pharmacy, University of Siena, Siena, Italy; 11 Department of Molecular and Translational Medicine, Pathology Unit, University of Brescia, Brescia, Italy; 12 Advanced MRI Center, University of Massachusetts Medical School, Worcester, MA USA; 13 Neuroimaging Research, Boston Children’s Hospital, Boston, MA USA

**Keywords:** EAE monophasic model, Neuroinflammation, TSPO-PET, MRI, Multiple sclerosis

## Abstract

**Background:**

Positron emission tomography (PET) using translocator protein (TSPO) ligands has been used to detect neuroinflammatory processes in neurological disorders, including multiple sclerosis (MS). The aim of this study was to evaluate neuroinflammation in a mouse MS model (EAE) using TSPO-PET with ^18^F-VC701, in combination with magnetic resonance imaging (MRI).

**Methods:**

MOG_35-55_/CFA and pertussis toxin protocol was used to induce EAE in C57BL/6 mice. Disease progression was monitored daily, whereas MRI evaluation was performed at 1, 2, and 4 weeks post-induction. Microglia activation was assessed in vivo by ^18^F-VC701 PET at the time of maximum disease score and validated by radioligand ex vivo distribution and immunohistochemistry at 2 and 4 weeks post-immunization.

**Results:**

In vivo and ex vivo analyses show that ^18^F-VC701 significantly accumulates within the central nervous system (CNS), particularly in the cortex, striatum, hippocampus, cerebellum, and cervical spinal cord of EAE compared to control mice, at 2 weeks post-immunization. MRI confirmed the presence of focal brain lesions at 2 weeks post-immunization in both T1-weighted and T2 images. Of note, MRI abnormalities attenuated in later post-immunization phase. Neuropathological analysis confirmed the presence of microglial activation in EAE mice, consistent with the in vivo increase of ^18^F-VC701 uptake.

**Conclusion:**

Increase of ^18^F-VC701 uptake in EAE mice is strongly associated with the presence of microglia activation in the acute phase of the disease. The combined use of TSPO-PET and MRI provided complementary evidence on the ongoing disease process, thus representing an attractive new tool to investigate neuronal damage and neuroinflammation at preclinical levels.

**Electronic supplementary material:**

The online version of this article (10.1186/s12974-017-1044-x) contains supplementary material, which is available to authorized users.

## Background

Multiple sclerosis (MS) is the most common disabling neurological disease of young adults, affecting 2.3 million people worldwide and leading, in most cases, to severe and irreversible clinical disability [1]. Despite the increasing number and efficacy of novel therapeutic options, curative treatments for MS patients are lacking. This chronic inflammatory disorder is characterized by demyelination and axonal damage, culminating in the development of multifocal sclerotic plaques [2]. Different peripheral immune system cells, including T cells, B lymphocytes, and macrophages infiltrate into the CNS, eliciting neuroinflammation, oligodendrocyte death, and axonal damage [3]. Focal demyelinated plaques surrounded by regions expressing inflammatory markers and gliosis represent the core neuropathological features of MS. Lesions are located in multiple white matter [4] areas like the optic nerves, spinal cord, brainstem, cerebellum, periventricular white matter, and corpus callosum [5]. However, focal plaques have been described also in gray matter (GM) [6]. Axonal damage represents another pathological hallmark of MS and may occur independently of chronic demyelination [7]. Diagnosis of MS is based on clinical presentation of patients and instrumental data such as cerebro-spinal fluid (CSF) examination and magnetic resonance imaging (MRI) [8]. MRI procedures and pathological correlates have been standardized according to the recommendations indicated in McDonald criteria 2010, recently revised by the MAGNISM group [9]. MS lesions are defined using gadolinium-enhanced-T1 or T2-weighted MRI sequences. Despite the diagnostic power, conventional MRI is not able to fully describe the complex neuropathological modifications associated with MS or explain the biological meaning of abnormalities present in “normal appearing” GM or WM regions [10]. The use of positron emission tomography (PET) enables the in vivo quantification of selected biological processes depending on the radiopharmaceutical adopted. The isoquinoline carboxamide ^11^C-(*R*)*-*PK11195, as well as second generation radiopharmaceuticals targeting the 18 kDa-translocator protein (TSPO) expressed on microglial cells, allow in vivo imaging of brain inflammation in a number of neurodegenerative and neuroinflammatory disorders, including MS [11–14].

In MS, microglia activation seems to play a dual role, both favoring chronic inflammation and neurodegeneration through the modulation of T cells and release of reactive species, proteolytic enzymes, or other neurotoxic molecules [15, 16] and promoting re-myelination and oligodendrocyte differentiation [17]. Clinical studies showed increased ^11^C-(*R*)*-*PK11195 binding not only in Gd-T1-enhanced lesions or in T2 hyperintense areas of patients [12] but also in T1 hypodense regions or normal-appearing WM or GM [18, 19]. For these reasons, the combined use of MR and PET imaging could represent a unique tool to improve pathological and diagnostic characterization of MS [20].

TSPO ligands have been also used in the experimental autoimmune encephalomyelitis (EAE), a well-established animal model for MS [21] that displays several neuropathological features of MS, including microglial activation. In some of these studies, the distribution of TSPO radioligands has been cross-validated with post mortem immunohistochemistry analysis [22–25]. Despite the interest, preclinical data deriving from both TSPO-PET and MRI in rodent models of MS are lacking [25]. To explore the potential advantage of a multimodal imaging approach also in preclinical studies, here, we describe the combined use of ^18^F-VC701-PET, previously developed and validated in our facility [26], and Gd-T1 and T2 MR sequences in a murine EAE model [27]. This study allowed to obtain complementary results and to investigate novel diagnostic approaches, especially in light of the recent development of the new hybrid PET/MRI scanners [28].

## Methods

### Radiotracer

^18^F-VC701 was prepared with the automated synthesizer TRACERLAB _FXFN_ (GE Healthcare, Milan, Italy) as previously described. In brief, [^18^F]F^−^ was produced with the ^18^O(p,n)^18^F nuclear reaction by irradiation of [^18^O]water. After an anhydrification step, the labelling was performed with the precursor VC622 (3 mg) in anhydrous DMSO at 140 °C for 20 min. The reaction mixture was injected in semi-preparative HPLC for the purification. The fraction corresponding to ^18^F-VC701 was collected in sterile water and the product recovered by solid-phase extraction on pre-activated Sep-Pak tC-18 cartridge. ^18^F-VC701 eluted with ethanol (0.7 mL) and diluted with a saline solution (10 mL) for the final formulation. Radiochemical purity of ^18^F-VC701 > 98% was requested for mice experiments.

### Animals

C57BL/6J mice were purchased from Charles River. Age-matched female, 8- to 12-weeks-old, were used in all experimental procedures. Animal were maintained and handled in compliance with the institutional guidelines for the care and use of experimental animals (IACUC), which have been notified to the Italian Ministry of Health and approved by the Ethics Committee of the San Raffaele Scientific Institute (Prot. N. SK552/2012 D.lsg. 116/1992 and N. 722/2016-PR D.lsg. 26/2016).

### EAE model

**Fig. 1:**
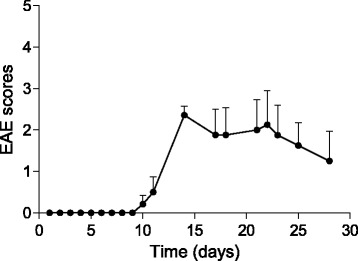
Clinical score of MOG_35-55_/CFA-immunized (EAE) mice. Graphical representation of clinical score of EAE mice evaluated daily after disease induction; mean scores ± SEM

C57BL/6J mice were immunized subcutaneously with 100 μg MOG_35–55_ (MEVGWYRSPFSRVVHLYRNGK, AnaSpec Inc), in incomplete Freud adjuvant (IFA, Sigma) containing 400 μg of heat-inactivated Mycobacterium tuberculosis H37 RA (Difco) and received two intravenous injections (i.v.) of 350 ng of pertussis toxin (List Biological Laboratories Inc), on day 0 and day 2 after immunization. The effect of immunization was checked using a standard clinical scale ranging from 0 to 5 [29] as follows: 0, asymptomatic; 1, limp tail; 2, limp tail and hind limb weakness; 3, partial hind limb paralysis; 4, complete hind limb paralysis; 5, moribund state, death (Fig. 1).

### Study design

A total of 40 mice were included in this study. For EAE mice: 27 mice were immunized as described in the previous paragraph; one mouse did not manifest clinical signs and was excluded and one had a severe reaction and died after immunization. The remaining 25 EAE mice were included in the study: 15 animals were dedicated to in vivo and ex vivo diagnostic imaging and 10 to immunohistochemistry (IHC) analysis. In particular, seven mice were sacrificed in ex vivo VC701 biodistribution experiment at 14 days post-infection (p.i.); four mice performed the in vivo longitudinal MRI study at 7, 14, and 28 days. The same group of animals underwent a PET scan at day 14 p.i. These animals plus additional 3 performed an ex vivo study with [^18^F]VC701 at 30 p.i. Time-activity curve (TAC) study was performed on a separate mouse to define PET acquisition time frame.

For healthy controls, 15 control animals were also included, 9 animals for in vivo and ex vivo imaging and 6 for immunohistochemistry (IHC) analysis.

### MRI imaging

Disease progression was monitored through longitudinal MRI imaging at different time points after immunization, with T1-Gd and T2-weighted sequences using a small-animal dedicated 7 Tesla MR scanner (Bruker Biospec 30/70, Ettlingen, Germany) and a four-channel mouse-head dedicated phased-array coil.

EAE (*n* = 4) animals at 7, 14, and 28 days post-immunization and control mice (*n* = 3) were anesthetized via intra peritoneal (i.p.) injection of tribromoethanol (Sigma Aldrich S.r.l., Italy) at 1.7% (20 μg/g) concentration prior to intravenous (i.v.) administration of Gadobutrol (Gadovist®, Bayer S.p.A., Italy) 0.3 mmol/kg, based on animal weight, in order to assess cerebral lesions. The animals were positioned prone on the dedicated heated apparatus. The image acquisition was performed on coronal plane using the following sequence protocol:

- Spin Echo T1 (time of repetition 526 ms, echo time 8 ms, voxel size 100 × 100 × 700 micron);
- RARE T2 (time of repetition 3007 ms, echo time 36 ms, voxel size 100 × 100 × 700 micron).

The brain lesions’ volumes were quantified manually using the manufacturer’s software (Paravision 5.1, Bruker, Ettlingen, Germany).

### Ex vivo biodistribution and in vivo PET studies with the TSPO radioligand ^18^F-VC701

The time course of TSPO signal in EAE mice was evaluated by tissue sampling analysis at 14 (EAE, *n* = 7) and 30 days post-immunization (EAE, *n* = 7). EAE animals were compared with 9 aged match healthy animals. Animals were injected in the tail vein with 4.0 ± 0.9 MBq of ^18^F-VC701 and sacrificed 240 min later. This time frame was selected on the basis of previous kinetics data on rats [26] and on a brain kinetic study on one EAE mouse showing a slight but progressive increase of brain radioactivity concentration, particularly in the cerebellum that is the brain region more affected in EAE mice. The increase in ^18^F-VC701 uptake measured as percentages of the injected dose per gram and cerebellum to cortex ratio is maximum between 2 and 4 h p.i. (see Additional file 1: Figure S1). Plasma was separated by centrifugation and counted with a blood sample in a gamma counter (LKB Compugamma CS1282, Wallac). Immediately after sacrifice, discrete areas as cortex, striatum, hippocampus, cerebellum, cervical enlargement, thoracic, and lumbar spinal cord were sampled and washed with cold saline. Samples were placed in pre-weighed tubes and counted. Radioactivity concentrations were calculated as percentages of the injected dose per gram of tissue (%ID/g) and expressed as ratio between interested area and plasma (%ID/g/%ID/g).

Four EAE mice and 3 healthy controls that underwent the longitudinal MRI study were evaluated also with PET at the time of maximum MRI-based abnormalities, i.e., 14 days post-immunization. ^18^F-VC701 was i.v. injected in a tail vein (5.9 ± 0.7 MBq). Each mouse was anesthetized with isoflurane in air (induction, 4%; maintenance, 2%) 240 min later, positioned prone on the PET scanner (YAP-S-PET II, ISE, Italy) bed, and scanned for 30 min (six dynamics frames of 5 min each). For the EAE mouse that underwent kinetic for the definition of the better time frame of tracer study, we acquired brain from ^18^F-VC701 i.v. injection up to 240 min. After reconstruction, correction for injected dose, and radioisotope decay, PET images were quantified using dedicated phantom and co-registered with MRI for the analysis with PMOD 3.2v (PMOD Technologies Ltd., Switzerland) software. ^18^F-VC701 radioactivity concentration was calculated using region of interest (ROI) analysis. More specifically, circular macro-ROIs were hand-drawn on the forebrain, midbrain, cerebellum, cervical enlargement, thoracic and lumbar spinal cord, and muscle in the MRI images and then applied on the co-registered PET images on three consecutive transaxial slices. Radioactivity concentrations were expressed as a percentage of injected dose per gram (%ID/g) and divided for radioactivity concentration in muscle (%ID/g/%ID/g). The evaluation of the relative distribution of TSPO-PET and MRI (T1 post-gadolinium enhancing and T2 hyperintensities) abnormalities was performed by visual inspection of the co-registered brain images of the four EAE mice.

### Histology and immunohistochemistry

Mice (5 EAE and 3 control mice per time point) were perfused with PBS followed by 4% paraformaldehyde (PFA). Two-micrometer-thick paraffin-embedded tissue sections from brains and spinal cords have been used for either routine pathological evaluation by hematoxylin and eosin (H&E) staining and immunohistochemistry (IHC). The following primary antibodies were used: rabbit anti-Iba-1 (1:300, Wako), rabbit anti-GFAP (1:200, Dako), and rat anti-MBP (1:50, Chemicon, Thermo Fisher Scientific, USA). Immunostaining has been revealed by Real EnVision Rabbit HRP or Rat-on-Mouse HRP (Biocare Medical, USA) and diaminobenzidine (DAB, Dako, Agilent Technologies, Italy) and counterstained with hematoxylin. Digital images have been acquired by Olympus DP70 camera mounted on Olympus Bx60 microscope, using CellF Imaging software (Soft Imaging System GmbH, Olympus, Italy).

Quantification of neuropathological features, although present both in the brain and spinal cord sections, has been performed in the spinal cords since it is highly reproducible and well established in the literature [30]. The degree of inflammation was expressed as percentage of infiltrated area over the total spinal cord section and demyelination was evaluated by measuring the percentage of demyelinated area over the total white matter area for each spinal cord section. An average of 8–10 spinal cord sections for each mouse was evaluated. Macrophage infiltration/microglial activation have been evaluated by assigning a semi-quantitative score to Iba-1 immunostaining and graded as follows: 0, none; 1, low; 2, moderate; 3, high [30].

### Statistical analyses

Differences in radioactivity concentration between regional distribution of ^18^F-VC701 in EAE and control animals were evaluated using the Student *t* test, defining a statistical significance threshold of *p* < 0.05. Mann-Whitney test was performed for MRI data of brain lesion volume measured through T1-gadolinium-enhanced and T2-weighted images (Software: GraphPad Prism 5.0).

## Results

### Immunized mice showed motor signs and MRI abnormalities typical of EAE that peaked at 14 days post-treatment

**Fig. 2:**
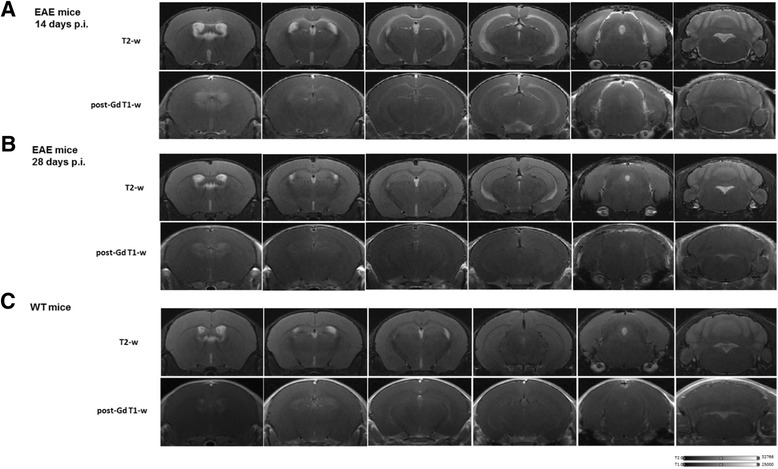
Magnetic resonance imaging (MRI) longitudinal study of a representative EAE mouse and healthy control. **a** Selected coronal MRI images acquired at 14 days p.i. showing focal lesions in T2-weighted and post-contrast T1-Gd-weighted images in different brain areas including hippocampus, corpus callosum, external capsule, and periventricular white matter. **b** Coronal T2 and T1-Gd MRI images acquired at 28 days p.i., representing the same brain coordinates of those shown in **a**. T2-enhanced regions are limited to the periventricular white matter and in the ventral aspect of hippocampus. EAE selected mouse exhibit 2.5 as clinical score at acute time point and 0 at late time point. **c** Selected coronal T2-weighted and post-contrast T1-Gd-weighted MRI images of a healthy control mouse

**Fig. 3:**
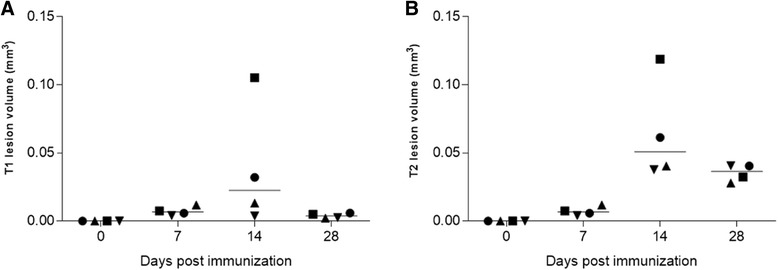
Magnetic resonance lesion volume measured in EAE mice brain. **a** T1-Gd-enhanced lesion volumes in four EAE mice measured at different time points after immunization. **b** T2 lesion volumes obtained in the same four EAE mice analyzed with T1-Gd MRI at different time points after immunization. Lesion volumes are expressed in millimeter cube and represented as single point including median; statistical analysis is performed using Mann-Whitney test

The vast majority of the immunized mice developed signs of EAE. The disease onset ranged approximately between day 10 and day 11 p.i. with a peak of clinical manifestations at 14–15 days p.i. One mouse did not manifest clinical signs, and one died after immunization. Despite a high variability of response, considering the whole group of mice, disease scores remained fairly stable over time (Fig. 1). At 7 days after disease induction, EAE mice T1 and T2 images showed no differences when compared to the control group, consistent with a previous report [27]. In the acute disease phase (14 days p.i.), lesions could be detected on both T2-weighted and post-contrast T1-weighted sequences in the regions including periventricular white matter, corpus callosum, thalamus, hippocampus, midbrain, pons, and cerebellum (Fig. 2a). At this time point, T2 hyperintense lesions overlapped with Gd-T1 enhancement in the majority of brain regions. Twenty-eight days p.i., signals detected by both T1/T2-weighted images decreased in the majority of regions (Fig. 2b; see panel (c) for control mouse). However, in the periventricular white matter and in the ventral aspect of the hippocampus, T2 alterations were still evident. Overall, these imaging data are consistent with the motor signs of immunized mice. The lesion volume measured on T1-weighted images at 14 days p.i. showed great variability (Fig. 3a), likely mirroring the known variability in the immunization. Belatedly (28 days p.i.), the T1-weighted lesion volume values decreased progressively to zero in all tested mice. The total brain lesion volume on T1-weighted images was lower when compared to that of T2-weighted images (Fig. 3b). T2-weighted images indeed showed the pattern of more concurrent events such as inflammation, edema, and demyelinating process, without however the possibility of differentiating them.

### An increase of regional uptake of ^18^F-VC701 and Iba-1 microglia/macrophage marker was present in both spinal cord and brain regions of EAE mice

^18^F-VC701 allowed detecting ex vivo and in vivo inflammatory reaction in the brain and spinal cord of EAE mice at the time of maximum increase of clinical signs. Mice were analyzed at 4 h after tracer injection to maximize signal to background ratio (see the “Methods” section).

**Fig. 4:**
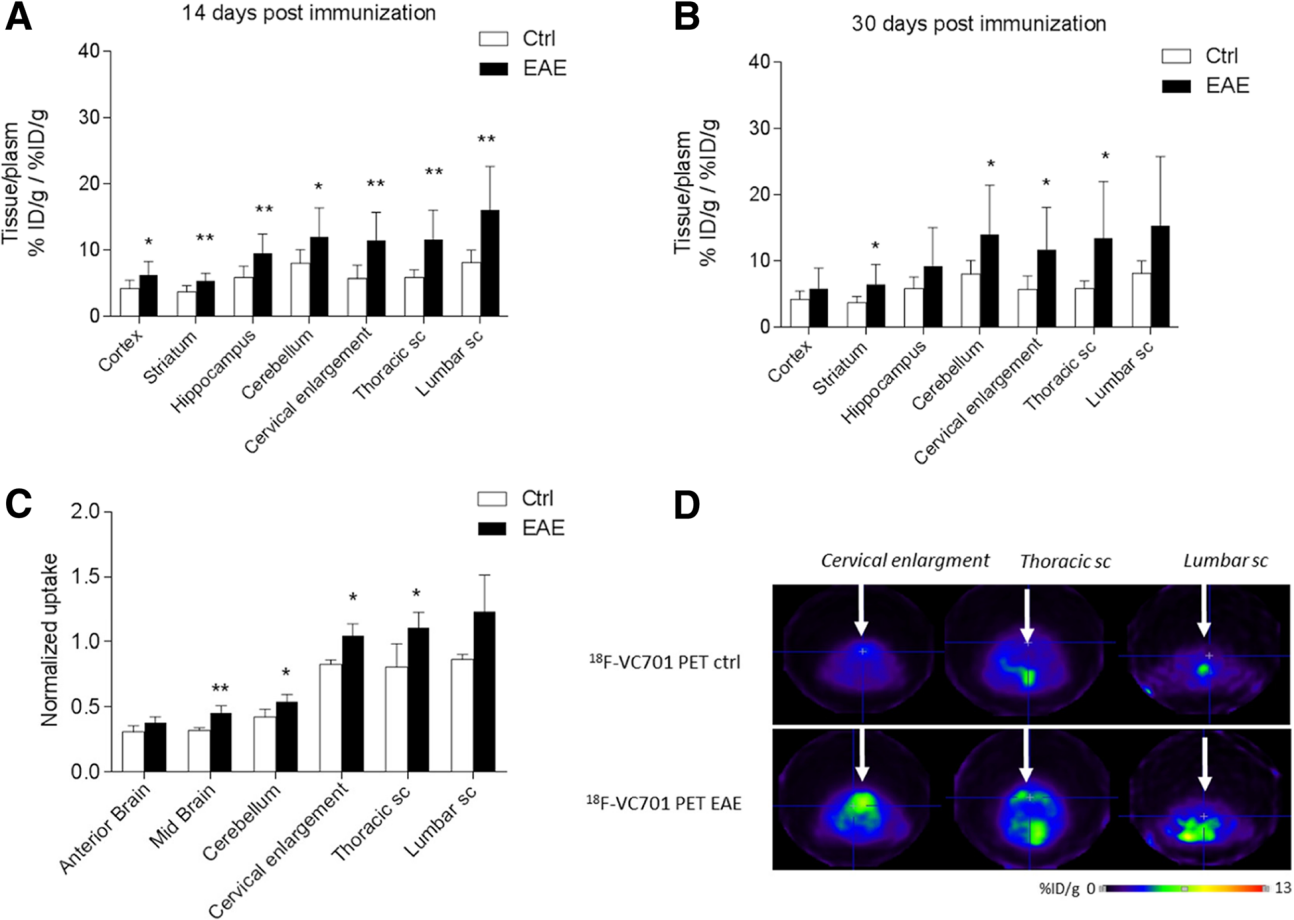
Ex vivo (**a**) and in vivo (**b**) distribution of ^18^F-VC701 in several regions of the central nervous system, in control and EAE mice. Regional distribution data are expressed as tissue to plasma ratios measured ex vivo at 14 (**a**) and 30 (**b**) days post-immunization. Regional distribution measured in vivo with PET at 14 days after immunization are expressed as tissue to muscle ratio (**c**); data are calculated as mean ± SD value; test *T*, *p < 0.05 vs controls. Representative PET images from the spinal cord of an EAE mouse with clinical score 2 at 14 days after immunization and healthy control mice injected with ^18^F-VC701 are shown (**d)**

Tissue sampling analysis revealed a significant increase of radioactivity concentration in the EAE mice as compared to control at the level of cortex and cerebellum (*p* < 0.05), and striatum, hippocampus, cervical enlargement, and thoracic and lumbar spinal cord (*p* < 0.01) at 14 days p.i. Radioactivity distribution progressed towards a cranio-caudal direction, with lower values in the cortex and higher values in the lumbar trait of the spinal cord (Fig. 4a).

At 30 days p.i., we observed a trend similar to that observed for the acute phase, although significant differences in ^18^F-VC701 uptake between EAE and WT (*p* < 0.05) only in the spinal cord and in discrete brain regions like the cerebellum.

**Fig. 5:**
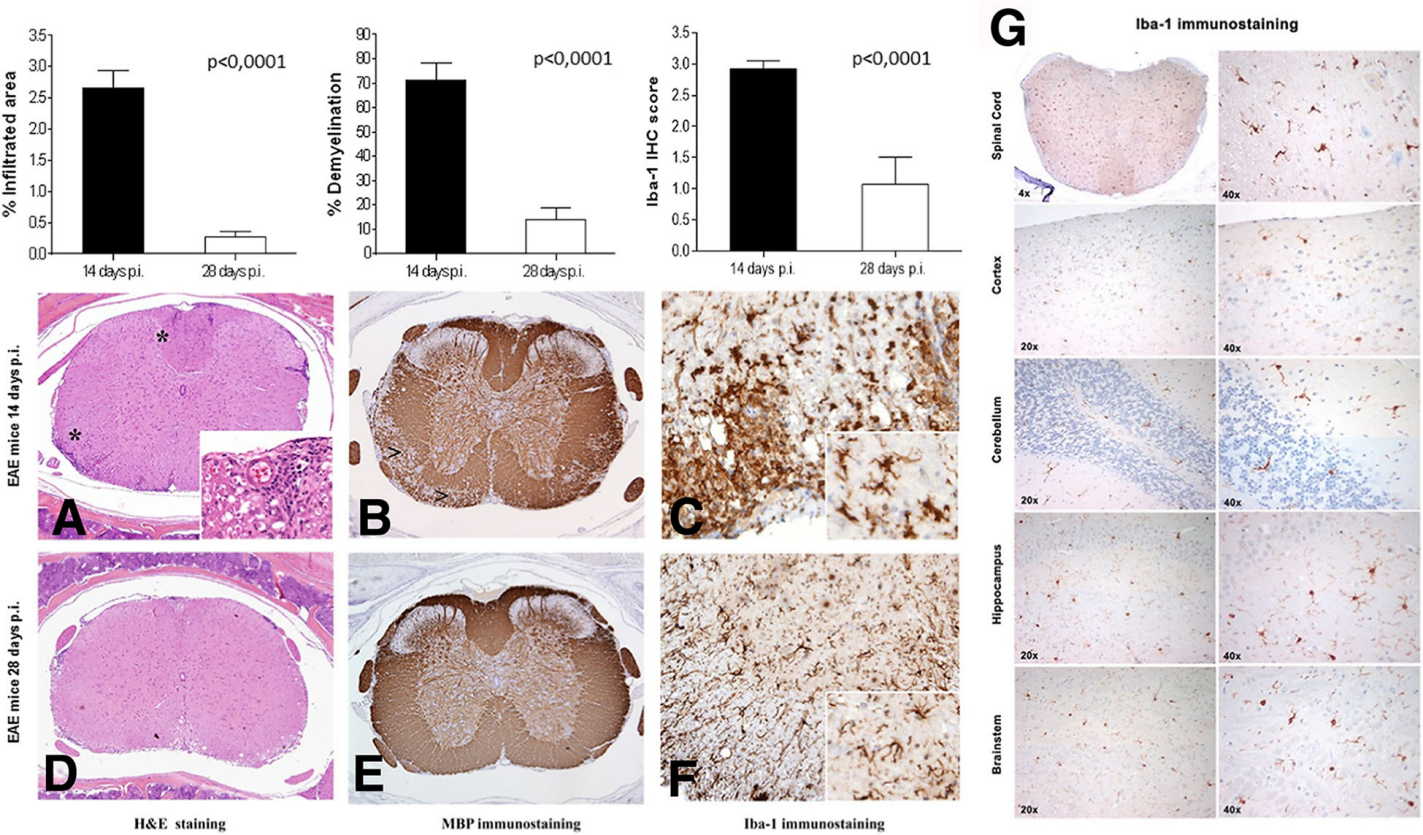
Histology and immunohistochemistry analysis. H&E staining of lumbar spinal cord sections of EAE mice sacrificed at 14 and 28 days p.i. (**a**, **d**; left graph). The degree of inflammation was higher during the acute phase of the disease (14 days p.i.), while barely detected in mice at 28 days p.i. Magnification shown in the inset from **a** revealed foci of inflammation (indicated by asterisks in the panel). Demyelination followed the same trend than inflammation passing from 14 to 28 days p.i. (**b**, **e**; middle graph), as indicated by arrows. At 2 weeks p.i. Iba-1 positive microglia and macrophage cells were highly represented, while scarcely detected at 4 weeks p.i. (**c**, **f**; right graph). Inset from **c** and **f** shows details on different microglia morphology during the two disease phases. Images **a**, **b**, **d**, and **e**, ×2 original magnification; images **c** and **f**, ×20 original magnification; insets, ×60 original magnification. Iba-1 positive staining images at different magnification (×20 and ×40) obtained from the cerebellum, cortex, hippocampus, and brain stem representative of one EAE mice sacrificed at 14 days p.i.. Iba-1 positive staining is visible in all brain regions but is particularly evident in cerebellum, brainstem, and hippocampus (**g**)

**Fig. 6:**
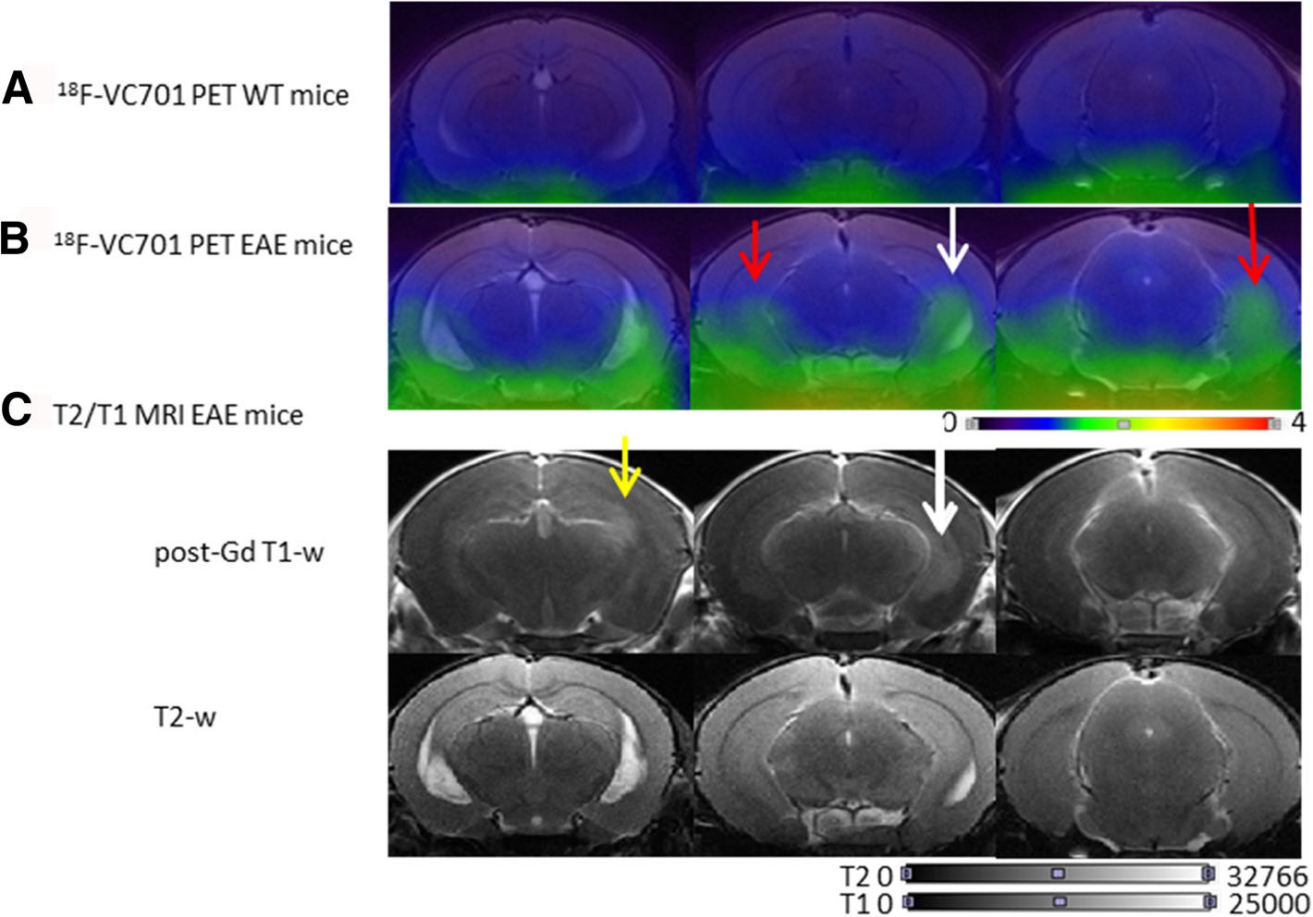
In vivo PET and MRI images representative of a healthy control and one of the EAE mouse (clinical score at 14 d.p.i. corresponding to 2 and 2.5 at 28 d.p.i.) evaluated at 14 days post-immunization. **a** ^18^F-VC701 PET and MRI co-registered coronal images of a healthy control; **b** ^18^F-VC701 PET and MRI co-registered coronal images from a EAE mice. **c** Corresponding MRI post-gadolinium T1-weighted (post-Gd T1-w) and T2-weighted (T2-w) images of the animal shown in **b**. Damaged brain areas are indicated by arrows. Some of these areas are overlapped in both PET and MRI (left hippocampus, white arrow); others are evident only in PET (bilateral hippocampi, red arrows) or in post-contrast MRI (yellow arrow) images. In the ventral part of coronal images of both control and EAE animals is present a marked region of radioactivity uptake deriving from non-specific accumulation of the tracer in extra-cerebral regions

In vivo PET analysis performed 14 days p.i. was consistent with the findings obtained from ex vivo experiments. Macro-ROI analysis showed a progressive increase of ^18^F-VC701 uptake towards cranio-caudal direction, with the highest increase of radiotracer uptake localized in lumbar trait of the spinal cord, and significant increase compare to controls observed in midbrain (*p* < 0.01) and cerebellum, cervical enlargement, and thoracic spinal cord (*p* < 0.05, Fig. 4c). Coronal and sagittal PET images of the three spinal cord traits considered are shown in Fig. 4d and Additional file 2: Figure S2, respectively. At visual inspection, EAE mice displayed heterogeneous clusters of increased radioactivity uptake in discrete brain regions including anterior cortex, midbrain, cerebellum, cervical enlargement and thoracic spinal cord when compared to control mice (see Figs. 4d and 6 and Additional file 2: Figure S2). Histopathological analysis of spinal cords and brains confirmed the association between the increased clinical score observed in immunized mice and the neuropathological features. Of note, at 2 weeks post-immunization, H&E staining revealed severe acute inflammation, particularly within the spinal cord, and preferentially distributed along the meninges and in perivascular areas (Fig. 5a and left graph). Myelin damage (Fig. 5b) and reactive gliosis (data not shown) were in line with the clinical score. However, during the chronic phase (at 4 weeks post-immunization), inflammation was barely detected (Fig. 5d), whereas demyelination, when quantified on myelin basic protein (MBP) immune-stained sections, was significantly lower as compared to control mice at 2 weeks p.i. (Fig. 5e and middle graph), mainly due to inflammatory clearance and re-myelination. This data mirrored the clinical scores as well. Of note, GFAP immunostaining highlighted diffuse and intense gliosis (data not shown). Accordingly, immunostaining for the microglia/macrophage-specific marker Iba-1 showed that activated microglia and macrophages were highly represented in the spinal cord at the peak of the disease (2 weeks p.i.), while during the chronic phase (4 weeks p.i.), EAE mice showed a significant reduction of the macrophage/microglia infiltration (Fig. 5c, f; right graph). Activated microglia show a typical hypertrophic morphology (inset from panel (c)), while at 4 weeks p.i. microglia shows the classical feature of resting cells with thin ramification and scarce cytoplasm (inset from panel (f)). Iba-1 staining at 14 days revealed the presence of microglial cells/macrophage infiltration in different brain regions including the brain stem, the cerebellum, the hippocampus and the cortex (Fig. 5g). According to the present converging PET, MRI, and ICH data, inflammation and tissue damage were more severe in the spinal cords than in the brain and followed a cranio-caudal gradient, with the lumbar spinal cord sections being the more affected.

### ^18^F-VC701-PET and MRI showed partially overlapping but complementary results in EAE mouse brain

PET signals in EAE mice only partially co-localized with damaged brain regions detected with MRI. In one mouse, PET evidence and clinical assessments were negative despite the presence of lesions on T2-weighted images. On the contrary, other mice showed highly concordant PET- and MRI-based evidence in multiple brain regions, i.e., hippocampus (3/4), hypothalamus (2/4), midbrain (3/4), corpus callosum (2/4) pons, and cerebellum both (4/4). However, and particularly for the hippocampus, the distributions of PET and MRI alterations were not completely overlapped (14 days p.i., Fig. 6, Additional file 3: Figure S3).

Otherwise, we observed clusters of significant ^18^F-VC701 uptake in absence of MRI T1 or T2 signals in other regions, such as in the striatum, cortex, and cerebellum. Lastly, no increases of radioactivity concentrations were detected in MRI-positive areas such as the thalamus and periventricular white matter (Table 1).

**Table 1:** Table summarizing lesions detected by visual inspection of co-registered brain images of the four EAE mice studied with PET/MRI performed at 14 days post-immunization

EAE lesion localization	EAE lesion detected by T2w MRI	EAE lesion detected by T1/Gd MRI	EAE lesion detected by ^F18^VC-701 PET
Periventricular white matter	4/4 **+**	4/4 **+**	0/4 **+**
Thalamus	1/4 **+**	1/4 **+**	0/4 **+**
Cortex	1/4 **+**	1/4 **+**	3/4 **+**
Striatum	2/4 **+**	2/4 **+**	3/4 **+**
Hypothalamus	2/4 **+**	2/4 **+**	2/4 **+**
Hippocampus	3/4 **+**	3/4 **+**	3/4 **+**
Corpus callosum	2/4 **+**	2/4 **+**	2/4 **+**
Periaqueductal gray matter	1/4 **+**	1/4 **+**	1/4 **+**
Midbrain	3/4 **+**	3/4 **+**	3/4 **+**
Pons	3/4 **+**	3/4 **+**	4/4 **+**
Cerebellum	1/4 **+**	1/4 **+**	4/4 **+**

The table indicates overlapping lesion in this two different imaging modalities on acute phase of the disease; +: detected

*T1/Gd* T1-weighted image with gadolinium contrast agent, *T2w* T2-weighted MRI, PET imaging with ^18^F-VC701 tracer

## Discussion

In the present study, we evaluated the feasibility of using ^18^F-VC701-PET to detect and follow microglia activation evolution in a monophasic MS mouse model obtained after MOG_35-55_ peptide treatment. Additionally, ^18^F-VC701 TSPO-PET was coupled with conventional T1-gadolinium-enhanced and T2 MRI imaging to evaluate the potential additive effect of combined PET and MRI techniques for the identification of EAE-related damages.

Clinical evaluation showed typical neurological deficits, starting around 11–12 days p.i. and peaking at 14 days p.i. In line with previous results from the same EAE animal model, we detected a slight and not significant reduction of motor signs at later disease phase [30]. MRI scans showed the presence of focal brain lesions in the acute phase of disease (i.e., at 14 days p.i.), detected on both T2-weighted and post-contrast T1-weighted sequences, in several brain regions, including the periventricular white matter, thalamus, corpus callosum, hippocampus, pons, and cerebellum. Of note, the maximum total lesion volume was observed on both post-contrast T1- and T2-weighted sequences at the same time point. At later disease phases, we observed a net reduction of T1 lesion burden, with T2- and T1-enhanced signals only partially overlapping, confirming the presence of demyelination, edema, or axonal damages also in the absence of inflammatory cell infiltration. As we previously observed, this discordance was particularly evident at later disease phase, where in the same brain regions we observed T2 hyperintensities in absence of any gadolinium uptake on T1-weighted images [27].

Both ex vivo tissue sampling and in vivo PET studies indicate that ^18^F-VC701 binding increases in different brain regions and in the spinal cord of EAE mice when macro-regions were used to quantify the signal. More specifically, using post mortem analysis, we found a maximum and significant increase of radioactivity concentration at 14 days p.i., with a regional distribution following a rostro-caudal positive gradient. A similar trend was present also when ^18^F-VC701 binding was quantified in vivo*.* However, PET data revealed a large heterogeneity in ^18^F-VC701 among animals, with cluster of increased radioactivity uptake localized in discrete brain regions. Post mortem tissue sampling studies indicated that, at 30 days p.i., a significant increase of radioactivity was present only in the striatum, cerebellum, and spinal cord; in these regions, the levels of radioactivity concentration were still high. The pattern of tracer uptake was in line with that described by other TSPO-PET studies performed with different radioligands and EAE models, showing a cranio-caudal increasing gradient [23]. Finally, also Iba-1 immunostaining measured post mortem was present both in the spinal cord and in the brain regions displaying higher uptake of ^18^F-VC701 [20].

To date, few imaging studies with TSPO ligands have been published in mouse and rat EAE models [22–24], and none of them combined conventional diagnostic MRI sequences with TSPO-PET. The only preclinical study associating TSPO-PET with MRI used T1-weighted sequences to focus on the longitudinal evaluation of ventricles volume enlargement, although not evaluating their relation with PET signal [25].

In our study, ^18^F-VC701 PET and MRI performed at the same day p.i. showed consistent but not overlapping results, confirming that also in the EAE model, the two image modalities may provide complementary information. In EAE animals, co-registered PET-MRI images showed TSPO signals also in regions lacking focal MRI abnormalities, particularly in gray matter regions including cortex, striatum, and cerebellum. These results although based on a small cohort of mice are in agreement with what is observed in patients. The presence of TSPO-PET signal outside MRI lesions was first described by Banati et al. in 2000 and then confirmed in other clinical studies [12, 31].

As previously stated, T1 and T2 MRI signals have different biological correlates including blood-brain barrier (BBB) leakage, edema, demyelination, gliosis, necrosis, or peripheral inflammatory cell infiltration [9]. In particular, gadolinium enhancement in T1-weighted MRI images indicates the presence of leakage in endothelial BBB and is considered in clinical routine as a surrogate marker of peripheral cell infiltration into the brain and representative of active lesions. On the contrary, an increased signal in T2 reflects different pathological events, not limited to the identification of focal areas of inflammatory edema, but also including astrogliosis, demyelination, axonal damage, or subtle BBB disturbance not detected with gadolinium.

TSPO-PET specifically reflects the presence of activated glial cells [32] and mainly those belonging to monocyte lineage, that in MS are mainly represented by perivascular macrophages or resident microglia. Therefore, increases of TSPO-related signal may be present not only inside active plaques but also in distant regions, reflecting axonal damages and the consequent anterograde, retrograde, or trans-synaptic degeneration of nearby neurons or in other brain areas where microglia may play a role [12]. From PET-TSPO studies in patients, it is known that the highest concordance between TSPO-PET and MRI is around gadolinium-enhancing T1-weighted lesions, which contain infiltrating immune cells, or in T2 hyperintense areas [12]. However, intense ^11^C-PK11195 uptake are present also around T1-weighted hypointense lesions, the so-called black holes, during relapse or in progressive MS patients [33]. This observation, already described by Banati in 2000 [12], strongly indicates that PET-positive T1 black holes are linked with an inflammatory state, thus supporting the potential use of TSPO-PET as a marker of disease progression [33]. In addition, in MS patients, increased TSPO ligand binding has been described in normal appearing WM or GM regions or around the rim of chronic active T2 plaques. These findings confirm the potential independent role of microglial cells in MS and prompt the need to better explore the potential diagnostic power of TSPO-PET [19, 34].

The concordance or discordance of MRI- and PET-based abnormalities can be of particular relevance in some areas, for instance the hippocampus, which is involved in cognitive processes such as memory, known to be impaired in MS [10]. According to recent data, neurotoxic molecules released by activated immune cells might interfere with synaptic plasticity leading to cognitive dysfunctions that are typical not only in EAE mice but also in MS patients [16]. Of note, in both patients and animal models, neuroimaging or post mortem studies demonstrated the presence of hippocampal atrophy involving particularly the CA1 sub-region [33, 35–37]. In the hippocampus, according to some authors, soluble mediators released by microglia contribute to the degeneration of GABAergic interneurons which, in turn, could influence synaptic and cognitive function in the hippocampus of EAE mice [36, 38]. On the other hand, several reports indicated that activated resident microglia have a general role in neuronal injury during MS and EAE and that microglia neurotoxicity seems to involve glutamate-driven excitotoxicity [39, 40].

For these reasons, the possibility to measure in vivo regional microglia activation at single animal level and to differentiate active inflammation from axonal damage in critical regions involved in cognitive processes may be particularly relevant. However, limits due to the low spatial resolution in preclinical PET imaging together with the high heterogeneous distribution of lesions should be carefully considered when TSPO ligands are used to track treatment effects in in vivo quantitative studies in mice.

## Conclusion

In conclusion, our ex vivo findings indicate that (i) ^18^F-VC701 allows to measure the presence of activated immune system cells in the brain and spinal cord of EAE mice, (ii) results from in vivo TSPO-PET and MRI give complementary information also in preclinical model of MS and their combined use represents a fundamental tool to better characterize the complex pathophysiology of MS, and (iii) the heterogeneous distribution of lesions together with the limited spatial resolution of PET should be carefully considered when TSPO ligands are used to track treatment effects in in vivo quantitative studies in mice.

## Additional files

Additional file 1: Figure S1. PET and MRI images of EAE mice at 14 days p.i. In vivo PET and MRI representative images of three of the four EAE mice used for the in vivo imaging evaluation at 14 days post-immunization. The fourth animal is shown in Fig. 6. A) ^18^F-VC701 PET and MRI co-registered coronal images of Mouse 1 (clinical score at acute phase 2.5 and 0 at late stage); B) ^18^F-VC701 PET and MRI co-registered coronal images of Mouse 2 (clinical score 2 at 14 d.p.i. and 2.5 at 28 d.p.i.); C) ^18^F-VC701 PET and MRI co-registered coronal images of Mouse 3 (clinical score 1.5 in acute phase and 0 at late stage of the disease). (DOCX 41 kb)

Additional file 2: Figure S2. PET TAC of ^18^F-VC701 of one EAE mice at 14 days p.i. In vivo time activity curve (TAC) of ^18^F-VC701 obtained for one EAE mouse at 14 days p.i.. The animal was injected in a tail vein with 3.92 MBq of ^18^F-VC701 and the brain acquired starting from tracer injection up to 120 min (12 frames of 10 min) and for 15 min at the time of 240. After reconstruction, correction for injected dose and radioisotope decay, PET images were quantified using dedicated phantom and co-registered with a specific T2 MRI template for the analysis with PMOD 3.2v (PMOD Technologies Ltd., Switzerland) software. Automatic ROIs were drawn on co-registered images on cortex and cerebellum and concentration of radiotracer calculated on each frame and expressed as percentage of injected dose per gram (%ID/g). (DOCX 471 kb)

Additional file 3: Figure S3. ^18^F-VC701 PET images of control and EAE mice. In vivo ^18^F-VC701 PET images of a control (top) and an EAE mouse (bottom) evaluated at 14 days p.i.. White arrows indicate in coronal (left) and sagittal (right) image of each panel the spinal cord trait considered. (DOCX 735 kb)

## Abbreviations

BBB: Blood-brain barrier
CFA: Complete Freud’s adjuvant
CNS: Central nervous system
CSF: Cerebrospinal fluid
EAE: Experimental autoimmune encephalomyelitis
GFAP: Glial fibrillary acidic protein
GM: Gray matter
H&E: Hematoxylin and eosin stain
IBA-1: Ionized calcium-binding adapter molecule 1
MBP: Myelin basic protein
MOG: Myelin oligodendrocyte glicoprotein
MRI: Magnetic resonance imaging
MS: Multiple sclerosis
PET: Positron emission tomography
PTX: Pertussis toxin
TSPO: Traslocator protein
WM: White matter
WT: Wild type
