# Supplementary material for: 18F-VC701-PET and MRI in the in vivo neuroinflammation assessment of a mouse model of multiple sclerosis
Source: J Neuroinflammation. 2018 Feb 5;15:33. doi: 10.1186/s12974-017-1044-x (PMC5800080; doi:10.1186/s12974-017-1044-x)
Supplement: Supplementary file 1 — PET and MRI images of EAE mice at 14 days p.i. In vivo PET and MRI representative images of three of the four EAE mice used for the in vivo imaging evaluation at 14 days post-immunization. The fourth animal is shown in Fig. 6. A) 18F-VC701 PET and MRI co-registered coronal images of Mouse 1 (clinical score at acute phase 2.5 and 0 at late stage); B) 18F-VC701 PET and MRI co-registered coronal images of Mouse 2 (clinical score 2 at 14 d.p.i. and 2.5 at 28 d.p.i.); C) 18F-VC701 PET and MRI co-registered coronal images of Mouse 3 (clinical score 1.5 in acute phase and 0 at late stage of the disease). (DOCX 41 kb) [file 12974_2017_1044_MOESM1_ESM.docx]

**Figure S1**


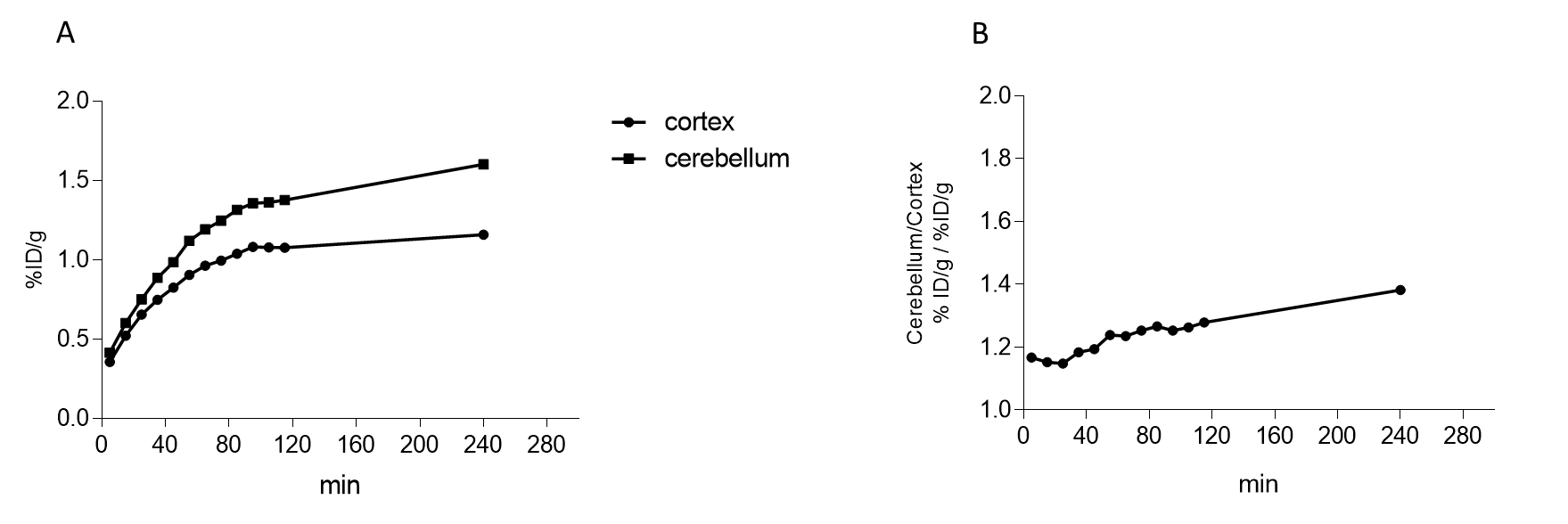


**Figure S1.** *In vivo* time activity curve (TAC) of ^18^F-VC701 obtained for one EAE mouse at 14 days p.i.. The animal was injected in a tail vein with 3.92 MBq of ^18^F-VC701 and the brain acquired starting from tracer injection up to 120 minutes (12 frames of 10 minutes) and for 15 minutes at the time of 240. After reconstruction, correction for injected dose and radioisotope decay, PET images were quantified using dedicated phantom and co-registered with a specific T2 MRI template for the analysis with PMOD 3.2v (PMOD Technologies Ltd, Switzerland) software. Automatic ROIs were drawn on co-registered images on cortex and cerebellum and concentration of radiotracer calculated on each frame and expressed as percentage of injected dose per gram (%ID/g).
