# Supplementary material for: 18F-VC701-PET and MRI in the in vivo neuroinflammation assessment of a mouse model of multiple sclerosis
Source: J Neuroinflammation. 2018 Feb 5;15:33. doi: 10.1186/s12974-017-1044-x (PMC5800080; doi:10.1186/s12974-017-1044-x)
Supplement: Supplementary file 2 — PET TAC of 18F-VC701 of one EAE mice at 14 days p.i. In vivo time activity curve (TAC) of 18F-VC701 obtained for one EAE mouse at 14 days p.i.. The animal was injected in a tail vein with 3.92 MBq of 18F-VC701 and the brain acquired starting from tracer injection up to 120 min (12 frames of 10 min) and for 15 min at the time of 240. After reconstruction, correction for injected dose and radioisotope decay, PET images were quantified using dedicated phantom and co-registered with a specific T2 MRI template for the analysis with PMOD 3.2v (PMOD Technologies Ltd., Switzerland) software. Automatic ROIs were drawn on co-registered images on cortex and cerebellum and concentration of radiotracer calculated on each frame and expressed as percentage of injected dose per gram (%ID/g). (DOCX 471 kb) [file 12974_2017_1044_MOESM2_ESM.docx]

**Figure S2**


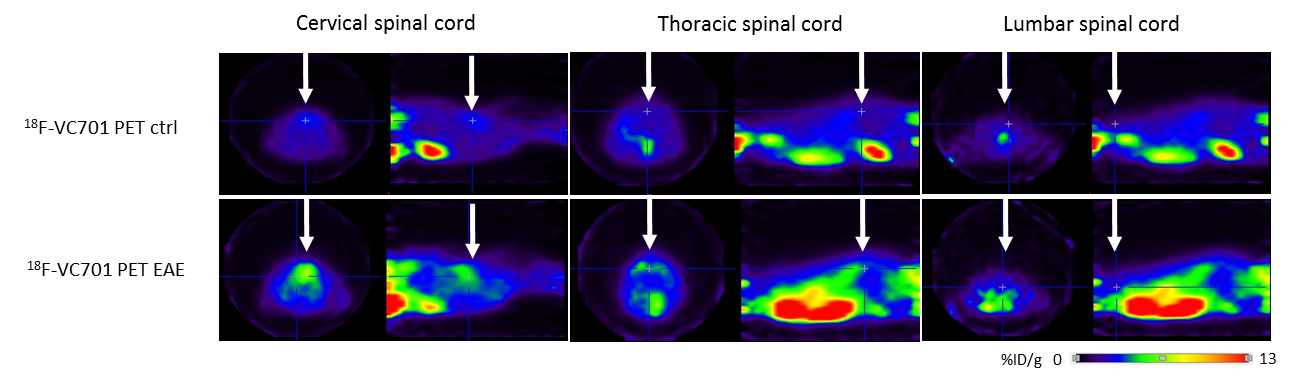


**Figure S2** *In vivo* ^18^F-VC701 PET images of a control (top) and an EAE mouse (bottom) evaluated at 14 days p.i.. White arrows indicate in coronal (left) and sagittal (right) image of each panel the spinal cord trait considered.
