# Supplementary material for: 18F-VC701-PET and MRI in the in vivo neuroinflammation assessment of a mouse model of multiple sclerosis
Source: J Neuroinflammation. 2018 Feb 5;15:33. doi: 10.1186/s12974-017-1044-x (PMC5800080; doi:10.1186/s12974-017-1044-x)
Supplement: Supplementary file 3 — 18F-VC701 PET images of control and EAE mice. In vivo 18F-VC701 PET images of a control (top) and an EAE mouse (bottom) evaluated at 14 days p.i.. White arrows indicate in coronal (left) and sagittal (right) image of each panel the spinal cord trait considered. (DOCX 735 kb) [file 12974_2017_1044_MOESM3_ESM.docx]

**Figure S3**


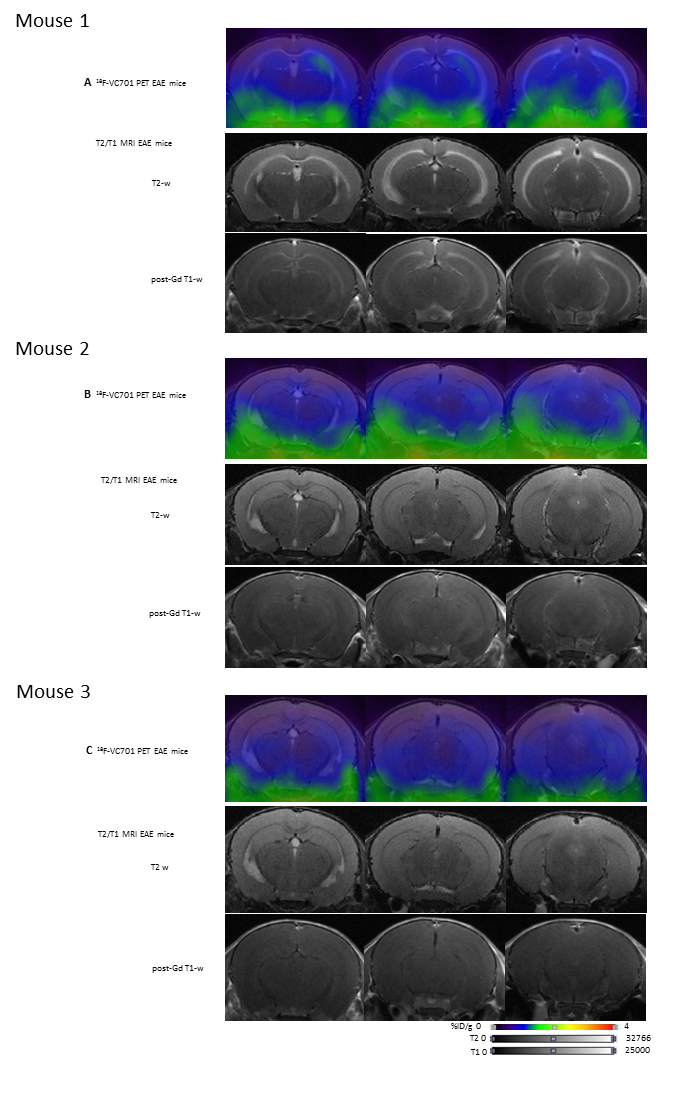


**Figure S3** *In vivo* PET and MRI representative images of three of the four EAE mice used for the *in vivo* imaging evaluation at 14 days post-immunization; the fourth animal is shown in Figure 6. A) ^18^F-VC701 PET and MRI co-registered coronal images of Mouse 1 (clinical score at acute phase 2.5 and 0 at late stage); B) ^18^F-VC701 PET and MRI co-registered coronal images of Mouse 2 (clinical score 2 at 14 d.p.i. and 2.5 at 28 d.p.i.); C) ^18^F-VC701 PET and MRI co-registered coronal images of Mouse 3 (clinical score 1.5 in acute phase and 0 at late stage of the disease).
